# Effects of Cerebrolysin® in Patients With Minimally Conscious State After Stroke: An Observational Retrospective Clinical Study

**DOI:** 10.3389/fneur.2019.00803

**Published:** 2019-08-02

**Authors:** Jun Yup Kim, Hyun Jung Kim, Hyo Seon Choi, So Young Park, Deog Young Kim

**Affiliations:** ^1^Department of Rehabilitation Medicine, Yonsei University College of Medicine, Seoul, South Korea; ^2^Research Institute of Rehabilitation Medicine, Yonsei University College of Medicine, Seoul, South Korea; ^3^Department of Rehabilitation Medicine, Nowon Eulji Medical Center, Eulji University, Seoul, South Korea

**Keywords:** Cerebrolysin, stroke, consciousness level, minimally conscious state, clinical study

## Abstract

**Introduction:** The neurotrophic drug Cerebrolysin is composed of low-molecular-weight peptides and amino acids and has been shown to have neuroprotective and neuroplastic properties. Cerebrolysin has been reported to promote the recovery of motor functions in central nervous system disorders; however, the effects on the consciousness improvements in post-stroke patients have not yet been studied extensively. Therefore, we aimed to examine the effectiveness of Cerebrolysin on improving the consciousness level of stroke patients with minimally conscious state (MCS).

**Materials and Methods:** In this retrospective study we included ischemic and/or hemorrhagic stroke patients with MCS according to the Coma Recovery Scale-Revised (CRS-R), who were admitted to our hospital between 2014 and 2017. All patients received comprehensive rehabilitation therapy including physical and occupational therapy. We compared patients treated with Cerebrolysin against patients who did not receive Cerebrolysin. Patients were included in the verum group if they received 10 mL of Cerebrolysin IV for at least 20 days. CRS-R scores were assessed at admission and discharge.

**Results:** Of 1,531 patients screened, 75 were included in the study (Cerebrolysin, *n* = 43; control, *n* = 32). Baseline characteristics were similar between groups. At discharge, ~2 months after onset of stroke, Cerebrolysin-treated patients improved significantly in the CRS-R (*p* = 0.010) after adjustment for confounders using linear mixed model (LMM), especially in the Oromotor (*p* = 0.003) and Arousal subscales (*p* = 0.038). No safety issues were observed.

**Conclusion:** This retrospective study suggests that Cerebrolysin may improve the level of consciousness in stroke patients with MCS, which should be further investigated in a well-designed, double-blind, placebo-controlled, randomized trial.

## Introduction

Stroke is the second leading cause of death in the world. About 14 million incidences of first-time strokes worldwide were reported in 2016 with 5.7 million deaths (1, 2), and stroke-related diseases, disorders, and pre-mature deaths are expected to double by 2035 (3). The social cost to the US economy due to stroke in the period from 2005 to 2050 is estimated at $2.2 trillion (4). Considering the tremendous socioeconomic burden, which is expected to further increase due to the continuous increase in life expectancy and proportion of elderly people, it is important to prevent or reduce stroke-related complications as early as possible. Neurologic complications of stroke include cerebral edema, hemorrhagic transformation, epilepsy, recurrent stroke, and consciousness disturbance. Though not always life-threatening, these can lead to delayed rehabilitation, long-term hospitalization, poor functional outcome, and increased health care costs. According to various stroke registry data, 4–38% of patients with stroke experience a decrease in the consciousness level or mental status (5). Studies have shown that there are possible risk factors for impairment of consciousness after stroke including age, sex, previous stroke history, atrial fibrillation, diabetes mellitus, alcohol consumption, stroke severity, anatomical location of injury site, massive cerebral infarct, and multiple brain infarcts (6).

Several reports have suggested that administration of growth hormones and neurotrophic factors such as brain-derived neurotrophic factor (BDNF), glial cell line-derived neurotrophic factor (GDNF), nerve growth factor (NGF), and ciliary neurotrophic factor (CNTF) may be associated with improved nerve regeneration after injury of the central nervous system (7). Cerebrolysin (EVER Neuro Pharma GmbH, AUSTRIA) is composed of low-molecular-weight peptides and amino acids and has been shown to exert neuroprotective and neurotrophic effects similar to endogenous neurotrophic factors (8).

Previous clinical trials in acute stroke with more than 1,500 patients enrolled have confirmed the safety and good tolerability of Cerebrolysin (9, 10). The CASTA study showed a significant decrease in mortality and disability as compared to placebo in severely affected patients (NIHSS > 12) (11). A recent meta-analysis of nine randomized controlled trials with Cerebrolysin has confirmed a clinically relevant and statistically significant effect of the drug in improving neurological function and clinical outcome after stroke (12).

Although there is robust evidence for Cerebrolysin to improve neuronal recovery after brain injuries, the effect of the drug on recovery of consciousness after severe stroke has not yet been studied. Accordingly, this retrospective study aimed to investigate the effects of Cerebrolysin on the recovery of consciousness in post-stroke patients with minimally conscious state (MCS).

## Materials and Methods

### Enrolment Criteria

In our study, we included adult, male, and female patients

- with confirmed stroke
- who met MCS criteria according to the Coma Recovery Scale-Revised (CRS-R) (13)
- treated with Cerebrolysin for at least 20 days; Cerebrolysin was administered intravenously at a daily dosage of 10 ml; patients who did not receive Cerebrolysin were allocated to the control group.
- with complete medical charts and CRS-R scores assessed at admission and discharge.

We did not include patients

- with a diagnosis of brain lesions other than stroke, such as traumatic brain injury (TBI) or hypoxic brain injury (HBI)
- with a pre-existing or active major neurological disease including TBI
- with progressive or unstable stroke
- with a history of significant alcohol or drug abuse and advanced liver, kidney, cardiac, or pulmonary disease (abnormal values of liver enzymes: total serum bilirubin >4 mg/dl, alkaline phosphatase > 250 U/L, SGOT/AST > 150 U/L, SGPT/ALT > 150 U/L, creatinine > 3.5 mg/dl)
- with a medical diagnosis of an expected survival of <1 year
- under treatment with traditional oriental medicine, vasodilators such as naftidrofuryl, cinnarizine, flunarizine, or nimodipine
- with any condition that would represent a contraindication for Cerebrolysin
- with a history of Cerebrolysin treatment
- who participated in other therapeutic studies.

All caregivers were informed about the risk and benefit of Cerebrolysin administration including possible serious adverse effects when the patient met the MCS criteria and then they decided whether to administer the Cerebrolysin to patients. The control group consisted of patients whose caregivers decided not to administer it to the patients.

The protocol of this study was approved by the Institutional Review Board (IRB) of the Yonsei University.

### Statistical Analysis

Medical record data were entered into a computer database and further analyzed with the Statistical Package for Social Sciences (SPSS Inc., Chicago, IL, USA) version 25. Data are expressed as mean ± standard deviation (SD), median ± interquartile range (IQR), and proportions, depending on the type of distribution of data. To compare baseline demographics and clinical data, χ^2^ tests and Fisher's exact tests were used for categorical variables and independent *t*-test was used for the parametric continuous variables, respectively. The variables that are already assumed to be normally distributed generally in the population group were regarded as parametric variables because the number of patients in each group exceeded 30. Paired *t*-test was used to compare parametric variables within the groups. The linear mixed model (LMM) was also used, setting the CRS-R total and subscores at admission and discharge as dependent variables and the other possible effectors as factors, to adjust the effects of covariates. Simple and multiple stepwise regression analyses were also used to determine the effects of covariates. A two-tailed *p* < 0.05 was considered statistically significant.

## Results

**Figure 1:**
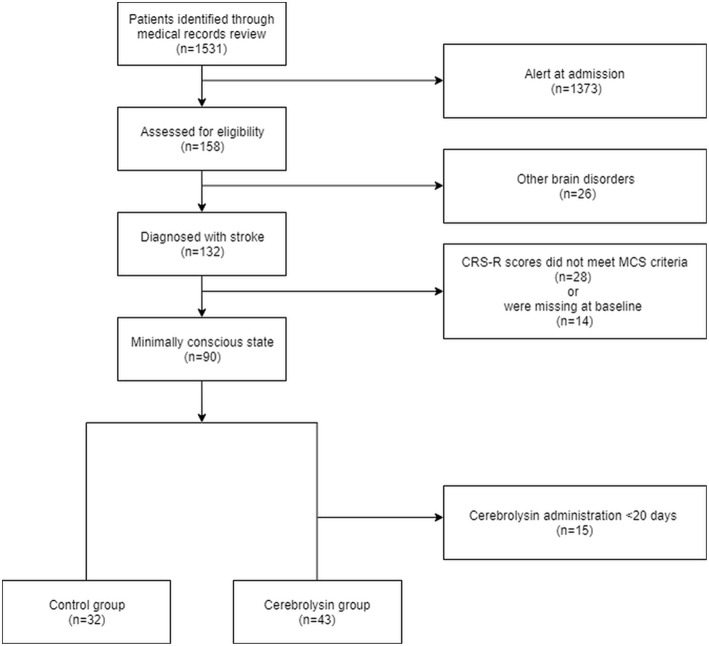
Flow chart of inclusion and disposition of subjects.

A total of 1,531 patients were identified through medical records review. Of these, 75 patients were included in our study, of which 43 (57.3%) patients were allocated to the Cerebrolysin group and 32 (42.7%) patients were allocated to the control group (Figure 1).

The age of the patients was 69.0 ± 14.9 (range, 23 to 93) years; 46 (61.3%) were males and 29 (38.7%) were females. Stroke was caused by intracerebral hemorrhage (*n* = 26), cerebral infarction (*n* = 45), and cerebral infarction with hemorrhage (*n* = 4). Further baseline characteristics are summarized in Table 1. There was no statistically significant difference in the baseline characteristics.

**Table 1:** Baseline characteristics of subjects.

**Characteristic**	**Cerebrolysin group** **(*n* = 43)**	**Control group** **(*n* = 32)**	***p*-value**
Age (years)	69.0 ± 15.4	68.7 ± 14.5	0.995
Sex (male/female)	23/20	23/9	0.106
Recurrence (first-ever/recurrent)	34/9	24/8	0.677
Etiology (infarction/hemorrhage/both)	18/19/6	19/9/4	0.296
CRS-R total scores at admission	13.1 ± 3.9	14.3 ± 3.1	0.151
Median CRS-R scores at admission (interquartile range)	
Total	14 (10.00–16.00)	15 (11.25–16.75)	
Auditory	2 (2.00–3.00)	2 (2.00–3.00)	
Visual	3 (2.00–4.00)	3 (2.00–4.00)	
Motor	5 (3.00–5.00)	5 (2.25–5.00)	
Oromotor	1 (1.00–2.00)	2 (1.00–2.00)	
Communication	1 (0.00–1.00)	1 (0.00–1.00)	
Arousal	2 (2.00–2.00)	2 (2.00–3.00)	
Length of hospital stay (days)	50.1 ± 13.7	64.0 ± 45.0	0.106

*Values are means ± standard deviation (SD). CRS-R indicates JFK Coma Recovery Scale-Revised*.

**Figure 2:**
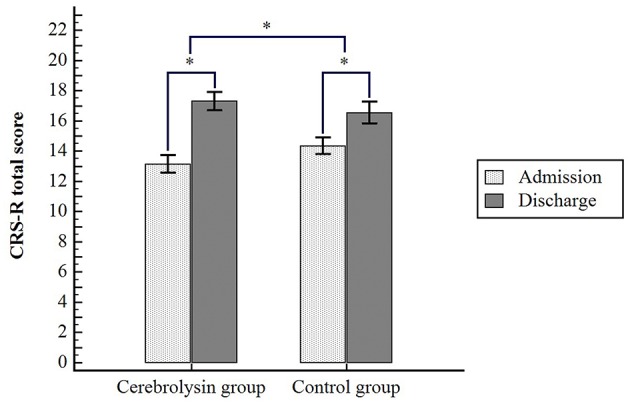
Changes of JFK-CRS total scores in both groups from admission to discharge. ^*^p < 0.05 comparing CRS-R total scores at discharge with scores at admission in each group by paired *t*-test, and comparing Cerebrolysin group vs. control group over time by linear mixed model (LMM).

In both groups, CRS-R total scores increased significantly between admission and discharge (*p* ≤ 0.001; Figure 2) by 4.2 points in the Cerebrolysin group and 2.3 points in the control group. In the inter-group analysis using LMM, the increase in the total score of CRS-R was statistically significantly higher in the Cerebrolysin group by the interaction of time and the use of Cerebrolysin (*p* = 0.010) after adjusting for age, sex, recurrence of stroke, length of hospital stay, duration from onset, concomitant use of neurostimulants, etiology of stroke, location of lesions, and laterality of lesions. CRS-R subscale analyses also showed higher improvement with use of Cerebrolysin in the Oromotor function (*p* = 0.003) and Arousal (*p* = 0.038; Table 2) after adjustment. A trend in favor of Cerebrolysin was also seen in the visual function subscale (*p* = 0.061). In the other subscales (auditory function, motor function, and communication), the improvement from admission to discharge was descriptively greater in the Cerebrolysin group. The use of Cerebrolysin was the only variable that showed significant explanatory power by multiple stepwise regression (*p* = 0.010) and other variables had no significant effects (Table 3).

**Table 2:** Comparison of CRS-R scores at admission and discharge between Cerebrolysin and control groups.

**CRS-R scores**	**Cerebrolysin group** **(*n* = 43)**		**Control group** **(*n* = 32)**		**Unadjusted** ***p*-value**	**Adjusted** ***p*-value^‡^**
	**Admission**	**Discharge**	**Admission**	**Discharge**		
Total score	13.1 ± 3.9	17.3 ± 4.0^†^	14.3 ± 3.1	16.5 ± 4.1^†^	0.010^*^	0.010^*^
Subscores						
Auditory	2.2 ± 0.8	2.9 ± 1.0^†^	2.4 ± 0.9	2.8 ± 1.0^†^	0.116	0.116
Visual	2.8 ± 1.1	3.9 ± 0.9^†^	3.2 ± 1.0	3.8 ± 1.1^†^	0.061	0.061
Motor	4.0 ± 1.5	4.9 ± 1.2^†^	4.0 ± 1.5	4.6 ± 1.6^†^	0.300	0.300
Oromotor	1.5 ± 0.6	2.1 ± 0.8^†^	1.8 ± 0.6	1.9 ± 0.7	0.003^*^	0.003^*^
Communication	0.5 ± 0.5	1.0 ± 0.7^†^	0.7 ± 0.5	1.0 ± 0.6^†^	0.351	0.351
Arousal	2.1 ± 0.7	2.5 ± 0.6^†^	2.3 ± 0.5	2.4 ± 0.5	0.038^*^	0.038^*^

*Values are means ± standard deviation (SD). CRS-R indicates Coma Recovery Scale-Revised*.

Bold values indicates

* *p < 0.05 comparing Cerebrolysin group vs. control group over time by linear mixed model (LMM)*.

† *p < 0.05 comparing subscores at discharge with scores at admission in each group by paired t-test*.

‡ *Adjustment for confounders including age, sex, recurrence of stroke, length of hospital stay, duration from onset, concomitant use of neurostimulants, etiology of stroke, location of lesions, and laterality of lesions was done using LMM*.

**Table 3:** Results of simple and multiple regression analyses of CRS-R improvement in all subjects.

**Variables**	**Simple regression**		**Multiple stepwise regression**	
	**β Coefficient**	***p*-value**	**β Coefficient**	***p*-value**
Age	−0.024 ± 0.026	0.353		
Sex (male)	0.241 ± 0.792	0.762		
Recurrent stroke	−0.566 ± 0.920	0.540		
Length of hospital stay	−0.022 ± 0.012	0.072		
Duration from onset	−0.004 ± 0.002	0.078		
Use of Cerebrolysin®	−1.975 ± 0.746	**0.010^§^**	−1.975 ± 0.746	**0.010^§^**
Dopaminergic drugs	−0.283 ± 0.859	0.743		
Methylphenidates	0.570 ± 0.885	0.522		
Acetylcholinesterase inhibitors	−1.363 ± 0.756	0.076		
Etiology of stroke				
Ischemia	Reference			
Hemorrhage	1.098 ± 0.829	0.190		
Both	−0.473 ± 1.180	0.690		
Location of lesions				
Supratentorial	Reference			
Infratentorial	−0.630 ± 1.106	0.571		
Both	0.518 ± 1.268	0.684		
Laterality of lesions				
Right	Reference			
Left	0.897 ± 1.771	0.614		
Bilateral	1.345 ± 1.763	0.448		

*Values are means ± standard error of the mean (SEM). CRS-R indicates Coma Recovery Scale-Revised*.

Bold values indicates

§ *p < 0.05 according to linear regression analysis*.

None of the patients experienced a serious adverse reaction (SAE) known to be related to the administration of Cerebrolysin.

## Discussion

Our retrospective study showed that Cerebrolysin significantly improved the level of consciousness of patients with MCS after acute stroke when compared to the control group. In our opinion, the improvement in the CRS-R score as observed in the Cerebrolysin group is clinically relevant and almost twice as high as in the control group. The increase in the total score of CRS-R was statistically significantly higher in the Cerebrolysin group by the interaction of time and the use of Cerebrolysin analysis even after adjustment for confounders using LMM. The use of Cerebrolysin was also significant in multiple regression analyses, and none of the potential confounding parameters had a significant influence on the outcome. Thus, Cerebrolysin treatment was the only effective factor in improving the CRS-R scores irrespective of age, sex, laterality of lesions, location of lesions, and the etiology of the stroke. We therefore consider our results very robust and interesting, and the results are also in line with a previous clinical Cerebrolysin study that showed improved cognitive functions in patients after ischemic stroke, albeit in a less affected stroke population (9).

A possible explanation of our findings is that treatment with Cerebrolysin might have increased the level of neurotrophic factors like BDNF. The brain of an individual with higher BDNF is more resistant to damage; thus, patients with higher BDNF levels may have higher capacity for consciousness recovery (14). Positive associations between plasma BDNF levels and cognitive functions like learning capacity, verbal delayed memory, abstract verbal reasoning, and processing speed were also shown in first episode psychosis (FEP) (15).

Furthermore, Cerebrolysin has been shown to interfere in several steps of the ischemic cascade and to promote neuroplastic processes *in vivo* and *in vitro* (16, 17), which also might have contributed to the observed improvement.

There are two representative circuits involved in the level of consciousness: the reticular activating system (RAS) and thalamocortical loops (TCL). Brain damage can result in dysfunctions of these neuronal circuits that interfere with consciousness (18). Consciousness depends on nerve impulses that can be influenced by neurotransmitters and their homeostasis. The most abundant and universal neurotransmitters in the brain are amino acids and monoamines. These include the oxygen-based amino acids such as glutamate and γ-aminobutyric acid (GABA), and monoamines such as dopamine, noradrenalin, adrenaline, and serotonin (19). Lack of awareness in the vegetative state (VS) and MCS is presumed to continue when restoration of neurotransmitter reserves remains incomplete in parts of the brain (20). Therefore, neurotransmitters like the oxygen-based amino acids and monoamines have been suggested as potential pharmacological agents for improvement of consciousness (19). In an open-label study performed with oral 10-mg zolpidem in 60 patients who suffered from impairment of consciousness, 20% of them showed improved behavior and/or CRS-R scores 1 h after administration, but significant improvements were observed in one patient only (use of functional objects) (21). Methylphenidate has been known to improve the action of catecholamines in the brain by blocking dopamine and norepinephrine reuptake by neurons. An effect of methylphenidate on the recovery of consciousness early in the post acquired brain injury period has previously been reported (22). Amantadine is one of the most commonly prescribed medications for patients with consciousness disturbance after brain injury (23) acting as indirect dopamine agonist with an *N*-methyl-D-aspartate antagonist (24). Amantadine was effective in three randomized trials dealing with patients suffering from consciousness disturbances after traumatic brain injuries (25–27). However, most of these studies were related to disturbances of consciousness after TBI rather than stroke. However, in these studies, no further improvement of the consciousness level was observed after drug discontinuation.

In our study, we did not find significant effects of dopaminergic drugs, methylphenidate, and acetylcholine esterase inhibitors. It is in line with a recent multicenter study to identify predictors of consciousness changes in 364 patients with disorder of consciousness after brain damage (28). They revealed that physical and cognitive treatments, age, and use of psycholeptic drugs were the significant predictors, but the use of antiparkinson drugs, psychoanaleptics, and muscle relaxants was not.

This study showed that the Oromotor and Arousal subscores were improved especially among the subscores. However, the underlying molecular mechanisms are still unclear. Based on neuroanatomy related to consciousness, we further suggest a hypothesis on underlying mechanism about the statistically significant difference in the amount of Arousal subscores' increments, which shows wakefulness, an important element of consciousness. RAS spreads from the midbrain with ascending projections to the basal forebrain complex (BFC) through two pathways, dorsally *via* the thalamus and ventrally *via* the hypothalamus (19). Previous studies have shown that NGF, one of the components of Cerebrolysin, plays an important role in survival and function of cholinergic neurons in the BFC (29). The BFC is a region of the brain responsible for attention, arousal, motivation, memory, and consciousness. In particular, NGF is fundamental for the functional integrity of cholinergic neurons in the central nervous system (30) and it also plays a role in regulating the phenotypic features of the noradrenergic nuclei of hypothalamus (31, 32). Thus, NGF contained in Cerebrolysin may have had a positive impact on the improvement of consciousness levels though enhancing two important components of the RAS circuit by upregulating the phenotypic features of the noradrenergic nuclei of hypothalamus and the function of cholinergic neurons of BFC. Although the level of consciousness may have been enhanced through the role of NGF, further basic research is needed to explain the underlying therapeutic mechanism of Cerebrolysin.

The findings of the present study have several limitations like the retrospective design and no control of concomitant use of neurostimulants at admission.

Even though this study showed that Cerebrolysin was effective in improving the level of consciousness after adjustment for confounding factors and multiple regression analysis showed no significant association of other drugs with the consciousness outcomes, we cannot fully exclude possible impacts since it was not possible to control potential interactions of the drugs or the impact of the dosage. In addition, the level of caregivers' attention to patient care and/or the patient's current severity might have affected the assignment to the control or experimental group, which could have acted as selection bias. Besides, patients diagnosed as VS were not included in the study because their number was too small to represent characteristics of VS patients. Furthermore, due to the small number of enrolled patients, the multivariate model might not have had enough power to adjust for more complex variables. Therefore, in future studies, the enrollment of more patients with longer follow-up periods should be considered. Also, we recruited only patients who had been given Cerebrolysin for 20 days or more and who had no contraindications to the drug. Therefore, we were not able to fully determine the safety profile of the drug or a dose relationship of the response to treatment.

In the future, prospective double-blind, randomized controlled studies including vs. patients controlling confounding factors mentioned above will be needed, and neuroimaging techniques such as resting state functional magnetic resonance imaging or positron emission tomography brain scan will be helpful in explaining the underlying mechanisms.

Since previous studies did not assess the level of consciousness as an outcome parameter, our results are therefore valuable in presenting a different view of the therapeutic value of Cerebrolysin. We consider our results very promising, in particular because we were able to achieve a significant improvement of the state of consciousness in a population of patients where meaningful clinical effects are rarely seen in clinical practice.

## Conclusion

The results of this study suggest that Cerebrolysin may have a positive effect on improving the level of consciousness in patients with post-stroke MCS. No. safety issues were observed for Cerebrolysin; thus, further investigations might be considered, which could include imaging techniques to examine also changes in functional connectivity and brain metabolism by Cerebrolysin.

## Data Availability

The datasets for this manuscript are not publicly available because the data is the property of the hospital that contains the patient's private information. However, at any time, an anonymous dataset can be provided via mail when the reviewer wishes. Requests to access the datasets should be directed to JK futurer22c@gmail.com.

## Ethics Statement

The protocol was approved by the Institutional Review Board (IRB) of the Yonsei University. Written informed consent was waived because this is a retrospective study. In order to protect vulnerable patients who couldn't express correctly due to the deterioration of cognitive function, the benefit and side effects of medication were explained to the caregiver prior to administration and only administered when the consent of the caregiver was obtained.

## Author Contributions

DK and JK envisioned the goals and design of this study. SP and HC collected clinical data of this study. JK organized the data of this study and used them for statistical analysis and interpretation. JK wrote the manuscript and conducted data analysis. DK and HK participated in the critical revision of this paper. DK conducted the final review and approved submission of the manuscript.

### Conflict of Interest Statement

The authors declare that the research was conducted in the absence of any commercial or financial relationships that could be construed as a potential conflict of interest.
